# Prolonged Remissions After Nivolumab Plus Gemcitabine/Oxaliplatin in Relapsed/Refractory T-cell Lymphoma

**DOI:** 10.1097/HS9.0000000000000672

**Published:** 2022-01-10

**Authors:** Roch Houot, Viola Poeschel, Bettina Altmann, Stephanie Angel, Lorenz Thurner, Thomas Illmer, Marc Andre, Martin Dreyling, Hervé Maisonneuve, Hervé Tilly, Stephanie Mayer, Olivier Casasnovas, Steven Le Gouill, Fritz Offner, Guillaume Cartron, Andrea Kerkhoff, Thomas Weber, Joerg Hoffmann, Marita Ziepert, Wolfram Klapper, Emmanuel Itti, Dirk Hellwig, Giorgi Natchkebia, Laurence de Leval, Andreas Rosenwald, Corinne Haioun, Laurent Dercle, Philippe Gaulard, Gerhard Held

**Affiliations:** 1 Department of Hematology, CHU Rennes, University of Rennes, INSERM U1236, Rennes, France; 2 Department of Hematology, Oncology and Rheumatology, Saarland University Medical School, Homburg/Saar, Germany; 3 Institute for Medical Informatics, Statistics and Epidemiology, University Leipzig, Germany; 4 Hematology-Oncology Practice, Dresden, Germany; 5 CHU UCL Namur, Yvoir, Belgium; 6 Department of Medicine III, LMU Hospital Munich, Germany; 7 Clinical Hematology, Centre Hospitalier Départemental Vendée, La Roche-sur-Yon, France; 8 Department of Hematology, Centre Henri Becquerel, Rouen, France; 9 Department of Internal Medicine III, Hematology and Oncology, University Hospital Regensburg, Regensburg, Germany; 10 Department of Hematology, CHU Dijon, Dijon, France; 11 Department of Hematology, CHU Nantes, Nantes, France; 12 University Hospital Ghent, Gent, Belgium; 13 Department of Hematology, CHU Montpellier, Montpellier, France; 14 Medizinische Klinik A, Hematology and Oncology, University Hospital Muenster, Germany; 15 Department of Internal Medicine IV, Hematology and Oncology, University Hospital Halle (Saale), Martin-Luther-University Halle-Wittenberg, Halle (Saale), Germany; 16 Department of Hematology, Oncology and Immunology, Philipps University Marburg, University Hospital Giessen and Marburg, Germany; 17 Institute for Medical Informatics, Statistics and Epidemiology, University Leipzig, Germany; 18 Department of Pathology, Hematopathology Section, University of Schleswig-Holstein, Campus Kiel, Germany; 19 Department of Nuclear Medicine, Henri Mondor University Hospitals, AP-HP/U-PEC, Créteil, France; 20 Department of Nuclear Medicine, University Hospital Regensburg, Germany; 21 Department of Internal Medicine 1, Westpfalz-Klinikum, Kaiserslautern, Germany; 22 Department of Laboratory Medicine and Pathology, Institute of Pathology, Lausanne University Hospital and Lausanne University, Switzerland; 23 Institute of Pathology, University of Würzburg, Germany; 24 Lymphoid Malignancies Unit, Henri Mondor University Hospital, APHP, Créteil, France; 25 Department of Radiology, Columbia University Irving Medical Center, New York, New York, USA; 26 Department of Pathology, Henri Mondor University Hospital, APHP, INSERM U955, Université Paris-Est, Créteil, France

Peripheral T-cell lymphomas (PTCL) represent a heterogeneous group of aggressive lymphomas, which usually carry a poor prognosis. Malignant T cells may overexpress programmed death ligand 1 (PD-L1), which signals via programmed death-1 (PD-1) receptor, and provides an inhibitory signal on normal T-cells further suppressing antitumor immunity. They can also express PD1, which may act as a tumor suppressor on malignant T cells.^1^ Thus, in principle, blocking the PD1/PDL1 synapse in PTCL may lead to either tumor regression or progression.^2^ Nevertheless, PD1-blockade demonstrated antitumor activity in monotherapy in patients with relapsed/refractory (R/R) PTCL with an ORR around 33%.^3,4^

The NIVEAU trial is an ongoing international, multicenter, randomized, open label, phase 3 study testing Nivolumab (Nivo) in combination with Gemcitabine and Oxaliplatin (Gem-Ox) [± Rituximab] for patients with aggressive (B and T cells) non-Hodgkin lymphoma, in first relapse or progression, who are not eligible for high-dose chemotherapy (NCT03366272). Here, we performed a preliminary analysis of the experimental arm (Nivo-GemOx) of the PTCL cohort to assess the safety and efficacy of this regimen in this population.

Key eligibility criteria include the following: first relapse or progression of PTCL, ineligibility for high-dose therapy (defined as age > 65 years or older than 18 years if HCT-CI score > 2), only 1 prior chemotherapy regimen including an anthracycline. Patients in the experimental arm were planned to receive 8 cycles Nivolumab (3 mg/kg at day 0 of cycle 1 and day 1 of cycle 2–8) plus Gemcitabine (1000 mg/m^2^ at day 1) and Oxaliplatin (100 mg/m^2^ at day 1) in 2-week intervals followed by additional 18 infusions of Nivolumab (3 mg/kg) biweekly as consolidation over a period of 1 year or until progression. Response was evaluated after 4 and 8 cycles of Nivo-GemOx according to the Lugano classification,^5^ and every 3 months by CT-scan during consolidation therapy. Each progression/relapse of PTCL had to be reported as an SAE.

The analysis (data cutoff October 16, 2020) included 12 patients enrolled in the experimental arm (Supplementary Digital Content Table 1, http://links.lww.com/HS/A216): 4 (33%) PTCL NOS, 3 (25%) AITL, 1 (8%) nodal TFH-PTCL, 2 (17%) ALCL ALK-, 1 (8%) EATL, and 1 (8%) MEITL. Median age was 69.5 years (range, 53–80), 7 (58%) patients were male, 2 (17%) had received a prior autologous stem cell transplantation, and 5 (42%) were refractory to first line therapy. At enrollment, performance status was 0–1 in 9 (75%) pts and 2 in 3 (25%) pts, 11 (92%) had Ann Arbor stage III–IV, 2 (17%) had B-symptoms, 7 (58%) had more than 1 extranodal site and 4 (33%) had elevated LDH. PD1 and PD-L1 were expressed by the tumor cells in 6/10 (60%) and 2/11 (18%) patients, respectively (Table 1).

**Table 1.** PD1 and PD-L1 Expression in Individual Patients^*a*^

Pt	Histology	PD1 Expression on Tumor Cells (%)	PD-L1 Expression on Tumor Cells (%)	Best Response by Investigator
1	Enteropathy-associated T-cell lymphoma (EATL)	0	0	PR
2	Peripheral T-cell lymphoma, NOS (PTCL-NOS)	5	0	CR
3	Anaplastic large cell lymphoma, ALK-negative (ALK-ALCL)	10	100	PR
4	Angioimmunoblastic T-cell lymphoma (AITL)	0	0	CR
5	Nodal peripheral T-cell lymphoma with TFH phenotype (TFH-PTCL)	0	0	PR
6	Peripheral T-cell lymphoma, NOS^*b*^	NA	NA	CR
7	Anaplastic large cell lymphoma, ALK-negative^*b*^	NA	50	PR
8	Angioimmunoblastic T-cell lymphoma	60	0	SD
9	Monomorphic epitheliotropic intestinal T-cell lymphoma (MEITL)	0	0	PD
10	Peripheral T-cell lymphoma, NOS	5	0	CR
11	Angioimmunoblastic T-cell lymphoma	50	0	PR
12	Peripheral T-cell lymphoma, NOS	90	0	PD

^*a*^PD-1/PD-L1 expression was assessed by IHC and centrally reviewed. Immunostains for PD1 and PD-L1 were performed using a Leica Bond automated immunostainer, with the following primary antibodies: PD1, clone NAT105 mouse monoclonal antibody, Abcam Ab52587; PD-L1, clone QR001 recombinant rabbit monoclonal antibody, Quartett.

^*b*^Absence or inadequate material for central pathology review.

NA = not assessable.

Patients have received a median of 6 (range, 1–8) cycles of GemOx and 8 (range, 1–26) infusions of nivolumab. Treatment was prematurely discontinued in 9 patients (7 during induction and 2 during consolidation), due to lymphoma progression (n = 6), toxicity (n = 2), or intercurrent disease (n = 1, yeast septicemia). There were 28 SAE in 11 patients, including 8 progressive diseases (Supplementary Digital Content Tables 2; http://links.lww.com/HS/A216 and 3; http://links.lww.com/HS/A216).

Nine (75%) patients achieved an objective response (4 complete responses and 5 partial responses). Two patients experienced primary progression upon Nivo-GemOx (Table 1): patient 9 (MEITL, PD1-negative) and patient 12 (PTCL-NOS, strongly PD1-positive). In these 2 patients, tumor growth rate (TGR) assessed during the first cycle of Nivo-GemOx (experimental period) was more than 2-fold higher than TGR assessed during the prior line of therapy (reference period) (Supplementary Digital Content Figure 1; http://links.lww.com/HS/A216). Unfortunately, the reference period cannot be a wash-out period (off-therapy) in aggressive lymphoma. In this fast-growing disease, the reference period is the prior line of therapy, as only on-treatment CT-scans are available before initiation of the experimental salvage treatment regimen. Thus, the standard criteria for hyperprogression, which have been established in solid tumors, could not be strictly applied in our cohort of patients.^6^ Nevertheless, patient 9 (MEITL, PD1-negative), which progressed after first-line therapy of 2 cycles of CHOEP and 2 cycles of high-dose Methotrexate experienced a second progression after the second cycle of GemOx. Patient 12 (PTCL-NOS, strongly PD1-positive), which progressed 1 month after 6 cycles of CHOP plus additive radiotherapy experienced a rapid second progression within its first cycle of study treatment, when again a partial remission lasting several months could be achieved after switching to third-line therapy. In this patient, nivolumab might have promoted lymphoma progression. However, shorter duration of remission in relapse represents a common phenomenon in lymphoma. Thus, establishing scientific criteria for hyperprogression are warranted. We did not find a clear correlation between PD1/PD-L1 expression on tumor cells and response to Nivo-GemOx. However, PD-L1 may also be expressed by bystander cells from the tumor microenvironment. This will be further explored in the final analysis of the clinical trial. Among the 9 responding patients, the median DOR was 14.9 months. Median PFS2 (time from randomization to second relapse/progression/death) was 6.9 months (95% CI, 0.3-13.5) versus 7.7 months (95% CI, 7.2-8.2) for PFS1 (time from diagnosis to first relapse/progression). Importantly, PFS2 was superior to PFS1 in 4 out of 10 patients (40%), and not informative in 2 pts: patient 10 who is still on therapy (ongoing PFS) and patient 8 who died prematurely of infection (Figure 1). Median OS was 24.8 months (95% CI, 1.6-47.9). After a median follow-up of 26.8 months, 7 patients have died, either from lymphoma (n = 5) or infection (n = 2, 1 COVID-19 infection and 1 yeast septicemia), and 5 remain alive.

**Figure 1.**
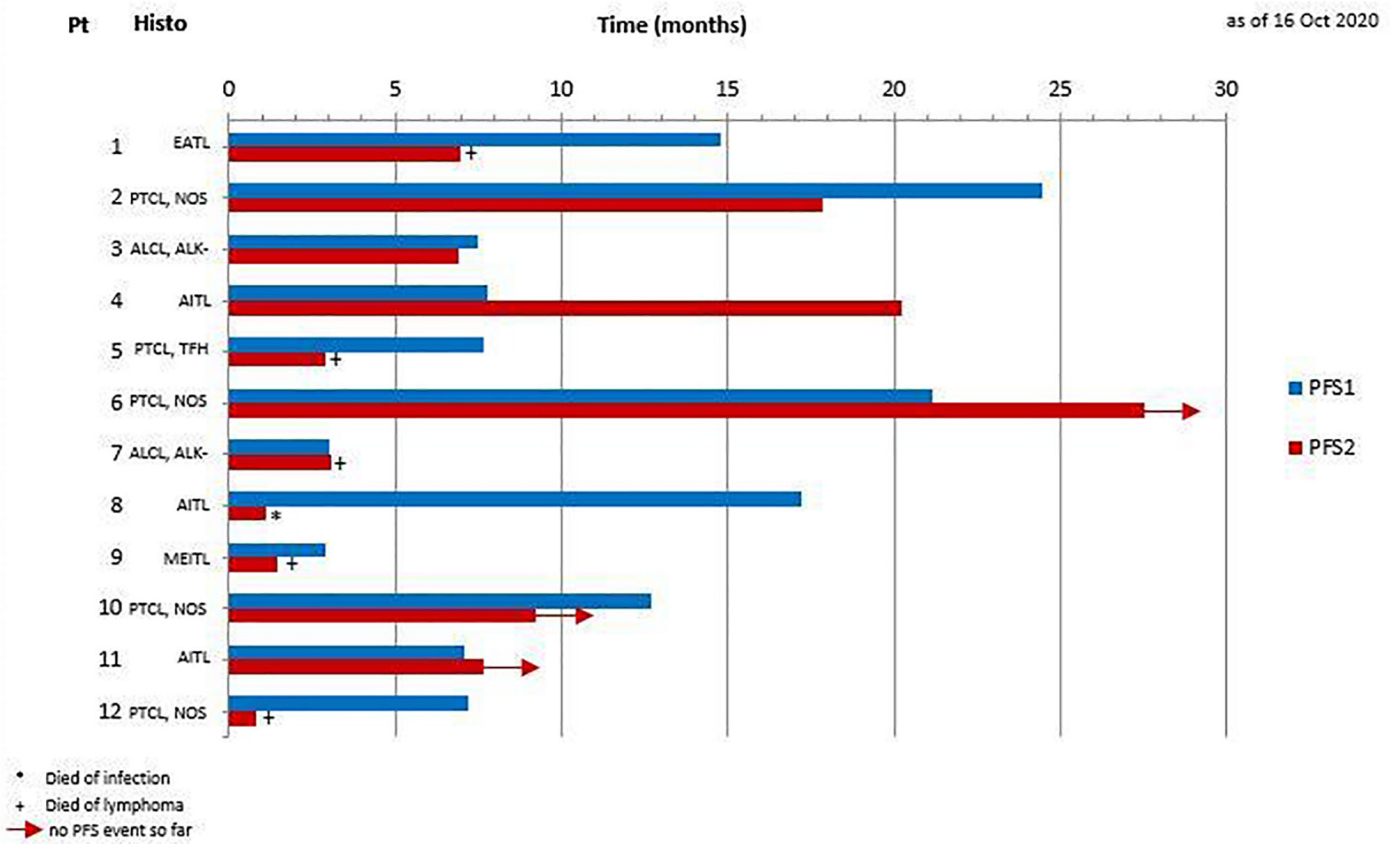
**PFS1 vs PFS2 for individual patients. PFS1 is defined as the time from diagnosis to first relapse/progression.** PFS2 is defined as the time from randomisation to second relapse/progression or death.

These preliminary results show that nivolumab in combination with GemOx is well tolerated. Although some reports raised the concern that anti-PD1 therapy may promote tumor proliferation in T-cell lymphoma,^1,7,8^ other studies demonstrated that anti-PD1 had efficacy in these neoplasms.^9–11^ Interestingly, the combination of Nivolumab with GemOx lead to high response rates and prolonged remissions (compared to first-line treatment) in a subset of patients. Longer PFS after salvage therapy suggests that it is more effective than the previous line of therapy, a CHOP-based regimen in our study. The combination of anti-PD1 antibody with chemotherapy may indeed have additive or synergistic clinical activity. Chemotherapy, notably gemcitabine and oxaliplatin, can promote tumor immunity by inducing immunogenic cell death and by disrupting the immunosuppressive tumor microenvironment.^12^ Additionally, anti-PD1 therapy may not only stimulate the antitumor immune response but also may sensitize to chemotherapy.^13–15^ In non-Hodgkin lymphoma, a retrospective study showed that the duration of response (DOR) to therapies given after checkpoint blockade therapy were longer than the DOR to treatment immediately prior to checkpoint blockade therapy, suggesting a potentiation effect of the immunotherapy.^14^ Cytostatic drugs like gemcitabine and oxaliplatin are disrupting DNA replication, which requires transition into cell cycle.^16,17^ One might speculate, that blocking the PD1 pathway results in increased T-cell receptor signaling and proliferation, rendering the cell more susceptible for chemotherapy.^1^

Overall, these preliminary results show encouraging efficacy and safety profiles of the Nivo-GemOx regimen in R/R PTCL. These findings will have to be confirmed on a larger number of patients and by comparing this combination with the control arm (Gem-Ox) once the NIVEAU study will be completed. Translational research (including evaluation of tumor microenvironment, oncogenic TCR alterations, and *Pdcd1* genomic deletions) will also be performed to identify predictive markers of efficacy. The NIVEAU phase 3 trial is actively enrolling patients.

## DISCLOSURES

R.H. received honoraria from Bristol-Myers Squibb, MSD, Gilead, Kite, Roche, Novartis, Janssen, and Celgene. V.P. received travel grants from Roche, Amgen, Abbvie. M.A. received advisory board from Takeda, Bristol-Myers-Squibb, Karyopharm, Gilead, Novartis, Seattle Genetics, Abbvie; research grants from Roche, Amgen, Johnson & Johnson, Novartis, Celgene; travel grants from Roche, Bristol-Myers-Squibb, Amgen, Celgene, Gilead. M.D. received research support (institution) Abbvie, Bayer, Celgene, Janssen, Roche; speakers honoraria from Bayer, Celgene, Gilead, Janssen, Roche; scientific advisory board from Astra Zeneca, Bayer, Beigene, Celgene, Gilead, Janssen, Novartis, Roche. H.T. received honoraria from Roche, Karyopharm, Aatra-Zeneca, Servier, Janssen-Cilag, BMS. S.M. did travel grants from Amgen, Abbvie; honoraria from Amgen, Novartis, Roche. G.C. received consultancy from Roche, Celgene; honorarium from Sanofi, Abbvie, Jansen, Gilead, Roche, Celgene. A.K. received travel grants from Roche and honoraria from Amgen, Novartis, BMS, and Takeda. T.W. received travel grants from Gilead, Roche, Takeda, honoraria from Roche and Takeda, research funding from Riemser and Takeda. D.H. received honoraria from Bayer Vital GmbH. C.H. received honoraria from Roche, Janssen-Cilag, Gilead, Takeda, Miltenyi and Servier and travel grants from Amgen and Celgene. P.G. received consultancy from Takeda, Gilead; research funding from Takeda, Innate Pharma; Sanofi Honoraria from Takeda; travel grants from Roche. G.H. received consultancy from Roche, BMS, MSD; research funding from BMS, Roche, Acrotech, Spectrum, Amgen; and travel grants from BMS, Roche. The NIVEAU is an ISR, which is financially supported by Bristol Myers Squibb with no influence on the content. All the other authors have no conflicts of interest to disclose.

## Supplementary Material


