# Supplementary material for: Prolonged Remissions After Nivolumab Plus Gemcitabine/Oxaliplatin in Relapsed/Refractory T-cell Lymphoma
Source: Hemasphere. 2022 Jan 10;6(2):e672. doi: 10.1097/HS9.0000000000000672 (PMC8751801; doi:10.1097/HS9.0000000000000672)

**Supplementary Tables**

**Supplementary Table 1. Patients’ characteristics.**

| Characteristics | Patients (n, %) |
| --- | --- |
| Median age, year (range) | 69.5 years (53-80) |
| Sex, n (%)  *Male*  *Female* |  |
|  | 7 (58%) |
|  | 5 (42%) |
| Baseline ECOG PS, n (%)  *0-1*  *2* |  |
|  | 9 (75%) |
|  | 3 (25%) |
| Prior Auto-SCT, n (%) | 2 (17%) |
| Prior Allo-SCT, n (%) | 0 |
| Stage of disease at enrollment  *I-II*  *III-IV* |  |
|  | 1 (8%) |
|  | 11 (92%) |
| >1 extra-nodal site at enrollment, n (%) | 7 (58%) |
| B-symptoms at enrollment, n (%) | 2 (17%) |
| LDH > ULN at enrollment, n (%) | 4 (33%) |
| Pathological subtype (primary pathologist), n (%)  *PTCL, NOS*  *AITL*  *PTCL, TFH type*  *ALCL, ALK-*  *EATL*  *MEITL* |  |
|  | 4 (33%) |
|  | 3 (25%) |
|  | 1 (8%) |
|  | 2 (17%) |
|  | 1 (8%) |
|  | 1 (8%) |

**Supplementary Table 2. Patients’ outcome.**

| Outcome | Patients (N=12) |
| --- | --- |
| No of GemOx cycles received (median, range) | 6 (1-8) |
| No of NIV cycles received (median, range) | 8 (1-26) |
| Premature treatment discontinuation* | 9 (7 during induction and 2 during consolidation) |
| Reasons for premature treatment discontinuation | 6 lymphoma progression, 2 toxicity and 1 intercurrent disease** |
| Grade 3-4 AEs | 12 |
| irAEs | 10 |
| Grade 3-4 irAEs | 4 |
| TRAEs | 11 |
| Grade 3-4 TRAEs | 10 |
| SAEs | 11 |
| Overall response | 9 (75%) (4 CR and 5 PR) |
| Primary progression | 2 (1 MEITL PD1-negative and 1 PTCL-NOS strongly PD1-positive) |
|  |  |
| Median PFS after Nivo-GemOx (PFS2) | 6.9 months (95% CI: 0.3-13.5) |
| Median OS* | 24.8 months (95% CI: 1.6-47.9) |

**After a median follow-up of 26.8 months, 7 patients have died, either from lymphoma (n=5) or infection (n=2, 1 COVID-19 infection and 1 yeast septicemia) and 5 remain alive; **Yeast septicemia*

**Supplementary Table 3. Significant adverse events**

| **Toxicity** |  | **Patients (N=12)** | |
| --- | --- | --- | --- |
| **Patients with AEs, Grade 3-4** | **12 (100%)** | | |
| **Patients with AEs, Events Grade 5** | **7* (58%)** | | |
| **Patients with AEs related to GemOx + Nivolumab** | **Grade 3-4** | | |
| **Total number of patients with AEs related to GemOx + Nivolumab** | **N=10/12** | | |
| **During induction** | **N=10/12** | | |
| Anemia | 4 | | |
| Thrombocytopenia | 8 | | |
| Leukocytopenia | 2 | | |
| Lymphocytopenia | 1 | | |
| Neutropenia | 3 | | |
| Leukocytosis | 1 | | |
| Fever | 1 | | |
| Infection | 1 | | |
| Peripheral polyneuropathy | 1 | | |
| Central nervous system vasculitis | 1 | | |
| Fatigue | 2 | | |
| Lipase increased | 3 | | |
| Gamma-glutamyltransferase increased | 2 | | |
| **During consolidation** | **N=2/6** | | |
| Diverticular perforation | 1 | | |
| Gamma-glutamyltransferase increased | 1 | | |
| **During follow up (n=5)** : no grade 3-4 adverse events related to GemOx + Niv have been reported | | | |
| **Patients with immune related AE** | **Grade 1-4** | | **Grade 3-4** |
| **Total number of patients with immune related AEs** | **N=10/12** | | **N=4/12** |
| **During induction** | **N=10/12** | | **N=4/12** |
| Cerebral vasculitis | **1** | | **1** |
| Diarrhea | 6 | | - |
| Rash | 2 | | - |
| Lipase increased | 7 | | 3 |
| Amylase increased | 6 | | - |
| Hypothyreodism | 1 | | - |
| **During consolidation** | **N=3/6** | | **N=0/6** |
| Diarrhea | 1 | | - |
| Lipase increased | 3 | | - |
| Amylase increased | 2 | | - |
| Arthralgia | 1 | | - |
| Hypothyreodism | 1 | | - |
| **During follow-up** | **N=1/5** | | **N=0/5** |
| Arthralgia | 1 | | - |
| Hypothyreodism | 1 | | - |

**5 patients due to lymphoma progression, 1 due to a yeast septicaemia, 1 due to a COVID-19 infection*

**Supplementary Figure 1. Two patients had two-fold increase in tumor growth rate (TGR) during Nivo-GemOx (experimental period) as compared to the previous line of therapy (reference period)**


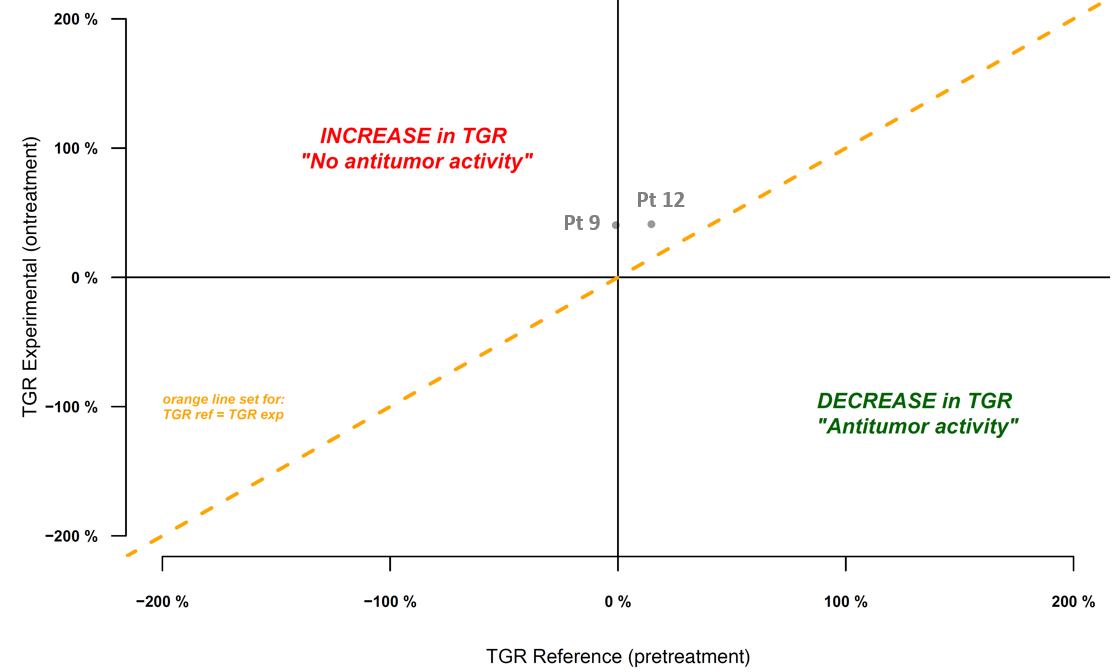

Supplement: Supplementary file 1 [file hs9-6-e672-s001.docx]
